# Contrast of oropharyngeal leak pressure and clinical performance of I-gel™ and LMA ProSeal™ in patients: A meta-analysis

**DOI:** 10.1371/journal.pone.0278871

**Published:** 2022-12-15

**Authors:** Yuan Tan, Jingyao Jiang, Rurong Wang

**Affiliations:** 1 Departments of Anesthesiology, West China Hospital of Sichuan University, Chengdu, China; 2 Laboratory of Anesthesia and Critical Care Medicine, Translational Neuroscience Center, West China Hospital, Sichuan University, Chengdu, China; University Hospital Knappschaftskrankenhaus Bochum, GERMANY

## Abstract

**Background:**

Conflicting outcomes have been reported for the i-gel™ and laryngeal mask airway (LMA) ProSeal™ in children and adults during general anesthesia. Randomized controlled trials (RCTs) that yielded wide contrast outcomes between i-gel™ and LMA ProSeal™ were included in this meta-analysis.

**Methods:**

Two authors independently identified RCTs that compared i-gel™ with LMA ProSeal™ among patients receiving general anesthesia by performing searches in EMBASE, Cochrane, PubMed, and ScienceDirect. Discussion was adopted to resolve disagreements. Data were counted with Review Manger 5.3 and pooled by applying weighted mean difference (MD) and rlsk ratio (RR), and related 95% confidence intervals.

**Results:**

A total of 33 RCTs with 2605 patients were included in the meta-analysis. I-gel™ provided a considerably lower oropharyngeal leak pressure [weighted average diversity (MD) = -1.53 (-2.89, -0.17), P = 0.03], incidence of blood staining on the supraglottic airway devices [RR = 0.44, (0.28, 0.69), P = 0.0003], sore throat [RR = 0.31 (0.18, 0.52), P<0.0001], and a short insertion time [MD = -5.61 (-7.71, -3.51), P<0.00001] than LMA ProSeal™. Compared with LMA ProSeal™, i-gel™ offered a significantly higher first-insertion success rate [RR = 1.03 (1.00, 1.06), P = 0.03] and ease of insertion [RR = 1.06 (1.01, 1.11), P = 0.03]. The gastric-tube-placement first insertion rate [RR = 1.04 (0.99, 1.10), P = 0.11], laryngospasm [RR = 0.76 (0.17, 3.31), P = 0.72], and cough [RR = 1.30 (0.49, 3.44), P = 0.60] between the two devices were similar.

**Conclusions:**

Both devices could achieve a good seal to provide adequate ventilation. Compared with the used LMA ProSeal™, the i-gel™ was found to have fewer complications (blood stainning, sore throat) and offers certain advantages (short insertion time, higher first-insertion success rate and ease of insertion) in patients under general anesthesia.

## Introduction

The common modality of airway administration in pediatric and adult patients for short surgical operations during general anesthesia is Supraglottic airway device (SAD) [1, 2]. Sufficient ventilation, delivery of anesthetic agents and oxygenation are provided with low-risk respiratory adverse events, displacing the demand for traditional tracheal intubation [3]. The second-generation SADs with a gastric drain tube have been recommended to decrease the danger of reflux and aspiration of the first-generation tools [4]. I-gel™ and LMA ProSeal™ belong to second-generation SADs.

Given the single-use supraglottic airway, i-gel™ shows a total insertion success rate of 100% with an anatomically designed and noninflatable mask made of a gel-like thermoplastic elastomer; a broadened and flattened stem with a hard bite block is adopted to decrease the axial rotation and malpositioning as a buccal stabilizer, and a port is provided for gastric tube interpolation [5]. The laryngeal mask airway (LMA) ProSeal™ is a laryngeal mask tool with an altered cuff and a drain tube. If inflated, its altered cuff presses the bowl of the tool forwards while improving the seal in virtue of the larynx [6].

To quantify the effectiveness of airway sealing and protecting airway in tools, oropharyngeal leak pressure (OLP) is adopted [7, 8]. Several randomized controlled trials (RCTs) have reported to compare i-gel^TM^ with LMA ProSeal™. Seven RCTs [9–15] observed higher OLP values in i-gel™ compared with LMA ProSeal™. However, 15 studies [16–30] recorded lower OLP values in i-gel™ compared with LMA ProSeal™, and 8 other research [3, 31–37] found no difference. Therefore, RCTs alone cannot sufficiently offer adequate insights into the clinical applications of i-gel™ and LMA ProSeal™.

To compare the superior airway sealing and certain advantages in patients under general anesthesia between the two SADs, 33 randomized controlled trials (RCTs) that yielded wide contrast outcomes between i-gel™ and LMA ProSeal™ were included in this meta-analysis. OLP was the primary result, and the first insertion success rate, insertion ease, intubation time, gastric-tube first insertion rate, and adverse events related to the SADs were the secondary results. In addition, subgroups analysis were performed in consideration of confounding elements, including age, type of operation, neuromuscular blocker (NMB) application, and the evaluation approach for OLP.

## Materials and approaches

The registration of meta-analysis was performed in PROSPERO (CRD42022312261), in inplasy.com (INPLASY2022100013) and on the foundation of the Preferred Reporting Items for Systematic Reviews and Meta-Analyses reports [38].

### Literature search

Eligible studies were made by searching e-databases EMBASE, Cochrane, PubMed, and the ScienceDirect. All studies were made in April 2022. The search items are shown below: (a) “i-gel™” and “i-gel™ laryngeal mask”; (b) “Laryngeal Mask Airway ProSeal,” “PLMA,” and “LMA ProSeal™”; (c) “random controlled trial,” “random,” and “randomly.” The pivotal words were connected applying “AND” (for “i-gel™,” “ProSeal Laryngeal Mask Airway,” and “randomized”) and “OR” (for “i-gel™” and “i-gel™ laryngeal mask”). The search was performed in English.

### Research selection

Only published prospective RCTs that compared i-gel™ with LMA ProSeal™ were included. Case reports, correspondence, reviews, manikin research, animal studies, and non-English articles were excluded.

### Data collection

The information below were gathered: the first author’s name, year of publication, the number of patients, age, type of operation, NMB application, premedication, mode of ventilation, evaluation approach for OLP, first-insertion success rate, ease of insertion, device insertion time, gastric-tube first-insertion success rate, and adverse events related to the SADs (sore throat, laryngospasm, blood-soiled devices, and cough). The information was collected by two independent authors (Yuan Tan and Jingyao Jiang). Discussion was adopted to resolve disagreements.

### Risk of bias evaluation

The risk of bias in RCTs was evaluated by using Cochrane collaboration standards. The criteria were as follows: randomization, concealment of allocation, blinding, incomplete data, selective reporting, and other bias. Each item was judged to be at high, unclear, or low risk of material bias.

### Statistical analysis

Data were counted with Review Manger 5.3 and pooled by applying weighted mean difference (MD) and rlsk ratio (RR), and related 95% confidence intervals. The random-effects model was applied if *I*^2^ >50%, which indicated high heterogeneity, and the fixed-effects model was used when *I*^2^<50%. Possible explanations for great heterogeneity were searched for with a sensitivity analysis. Subgroups were explored in consideration of confounding elements, including age, kind of operation, NMB application, and the promising role of the evaluation approach for OLP. Inspection of funnel plots (if the number of trials was beyond 10) was adopted to test the publication bias of including articles by visually.

## Results

**Fig 1:**
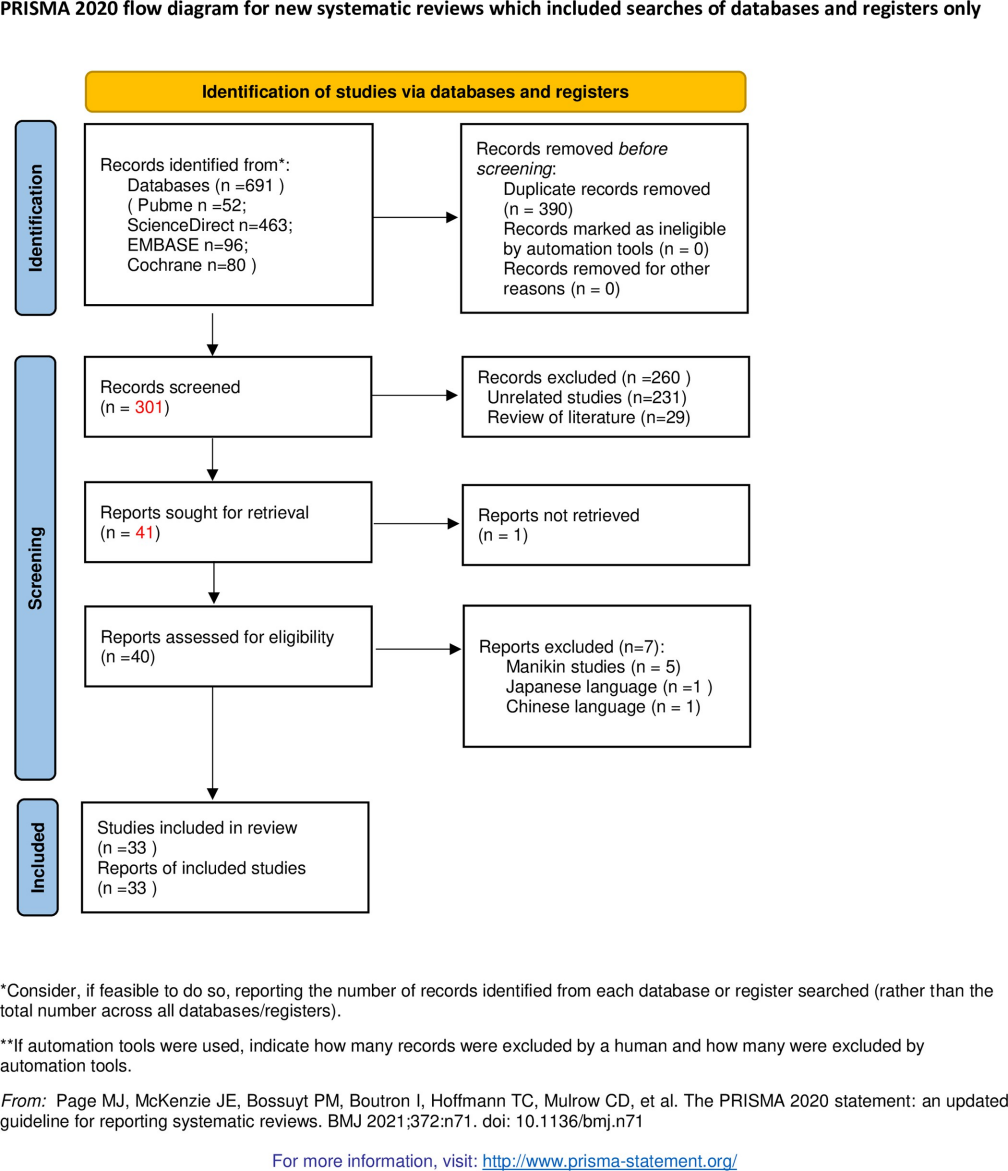
Flow chart of meta-analysis.

Fig 1 illustrates the particular procedures and research selection. The initial search yielded 691 articles (PubMed = 52, Embase = 96, ScienceDirect = 463, Cochrane Library = 80). After excluding duplications, 301 studies were examined. Next, 260 of the 301 studies were excluded because of unrelated studies and reviews. Apart from 1 not retrieved report, the remaining 40 studies were continued to be examined. Then, 7 of 40 studies were excluded based on the exclusion criteria. Finally, a total of 33 studies were included in this meta-analysis [3, 9–37, 39–41]. Tables 1 and 2 show the features and methodological quality of RCTs, respectively.

**Table 1:** Characteristics of included trials.

**Surgery**	**Premedication**	**NMB**	**Ventilation**	**OLP measuremen**
Elective hernioplasty, laparoscopic cholecystectomy, tibial plating, humerus plating and skin grafting	Midazolam 1mg IV	Rocuronium 0.9 mg/kg	Controlled	Audible leak
Elective gynaecological or orthopaedic surgery	Midazolam 0.05–0.1 mg/kg orally	No	Controlled	Manometer
Elective laparoscopic cholecystectomy	Ranitidine 50 mg IV	Vecuronium 0.08-0.1mg/kg IV	Controlled	Audible leak
Lower-extremity orthopaedic surgery	No	Rocuronium 0.6 mg/kg IV	Controlled	Manometer
Lower abdominal, inguinal and orthopedic surger	Midazolam 0.3mg/kg orally	No	Spontaneous	Manometer
Short stay elective surgery	Midazolam 0.5 mg/kg orally	No	Controlled	Manometer
Elective surgeries <1hour	Midazolam 0.5 mg/kg orally	No	Spontaneous	Manometer
Laparoscopic gynecologic operation	No	Rocuronium 0.6 mg/kg IV	Controlled	Manometer

**Surgery**	**Premedication**	**NMB**	**Ventilation**	**OLP measuremen**	**Author/Year**	**Age**	**Group**	**Number**
Elective surgeries of less than one hour duration	Midazolam 0.3mg/kg orally	No	Spontaneous	Manometer	Singh [17] 2009	adult	i-gel LMA-ProSeal	30 30
Elective surgery	No	No	Spontaneous	Audible leak	Gasteiger [18] 2010	19–70y	i-gel LMA-ProSeal	75 76
Elective surgery	Alprazolam 0.25 mg orally	Rocuronium 0.6 mg/kg IV	Controlled	Manometer	Sharma [19] 2010	adult	i-gel LMA-ProSeal	30 30
Elective surgery	Midazolam 0.5mg/kg orally	No	Not reported	Audible leak	Shin [20] 2010	adult	i-gel LMA-ProSeal	64 53
Elective surgery	Diazepam 5mg orally	Atracurium 0.5 mg/kg IV	Controlled	Not reported	Das [9] 2012	1-6y	i-gel LMA-ProSeal	30 30
Elective short surgical procedures	No	No	Spontaneous	Audible leak	Gasteiger [32] 2012	1.5-6y	i-gel	51 51
Elective short duration pediatric surgery	Midazolam 0.5mg/kg orally	Atracurium 0.5 mg/kg IV	Controlled	Audible leak	Goyal [10]	2-5y	i-gel LMA-ProSeal	40 40
Elective surgery	Midazolam 0.05 mg/kg intramuscular	Vecuronium 0.1 mg/kg IV	Controlled	Audible leak	Jeon [13] 2012	18-65y	i-gel LMA-ProSeal	15 15
Short surgical procedures	Midazolam 0.02 mg/kg IV	No	Spontaneous	Manometer	Mitra [11] 2012	5-10y	i-gel LMA-ProSeal	30 30
Elective surgery	Rectal 30 mg/kg paracetamol	No	Ventilated manually	Manometer	Van Zundert [37] 2012	18-80y	i-gel LMA-ProSeal	50 50
Elective procedures	Midazolam 7.5 mg orally	No	Controlled	Audible leak	Chauhan [21] 2013	18-65y	i-gel LMA-ProSeal	40 40
Elective surgeries	Midazolam 2 mg IV	Atracurium 0.5 mg/kg I.V	Controlled	Manometer	Fukuhara [31] 2013	3months-15y	i-gel LMA-ProSeal	67 67
Elective gynecological laparoscopic surgery	Midazolam 2 mg IV	Atracurium 0.5 mg/kg I.V	Controlled	Audible leak	Das [39] 2014	20-30y	i-gel LMA-ProSeal	30 30
Elective gynecological laparoscopic surgery	Midazolam 0.03 mg/kg IV	Rocuronium 0.6 mg/kg I.V	Controlled	Manometer	Kini [36] 2014	18-60y	i-gel LMA-ProSeal	24 24
Extra-ocular ophthalmic surgery	Midazolam 0.3 mg/kg orally	No	Controlled	Audible leak	Saran [33] 2014	1-12y	i-gel LMA-ProSeal	30 30
Elective surgeries	Alprazolam 0.25 mg orally	Vecuronium	Controlled	Manometer	Ekinci [40] 2015	18-65y	i-gel LMA-ProSeal	40 40
Elective surgeries	Midazolam 0.3 mg/kg oral	Atracurium 0.5 mg/kg IV	Controlled	Audible leak	Jadhav [22] 2015	18-60y	i-gel LMA-ProSeal	30 30
Elective superficial or peripheral surgery	No	No	Spontaneous	Audible leak	Kayhan [15] 2015	infants and neonates	i-gel LMA-ProSeal	25 25
Elective short surgical procedures	Midazolam 0.05 mg/kg IV	Vecuronium 0.1 mg/kg IV	Controlled	Audible leak	Henlin [24] 2015	>18y	i-gel LMA-ProSeal	99 98
Elective surgeries	No	Atracurium 0.5 mg/kg IV	Controlled	Audible leak	Mishra [26] 2015	18-65y	i-gel LMA-ProSeal	30 30
Elective surgical procedures	Alprazolam 0.25 mg oral	Rocuronium 0.6 mg/kg IV	Controlled	Manometer	Mishra SK [23] 2015	Adult	i-gel LMA-ProSeal	30 30
Elective short surgical procedures	No	No	Controlled	Audible leak	Mukadder [25] 2015	18-60y	i-gel LMA-ProSeal	35 35
Minor (<1 hour in duration) elective surgery	No	No	Controlled	Audible leak	Peker [34] 2015	1-10y	i-gel LMA-ProSeal	15 15
Elective surgery	Alprazolam 0.25 mg oral	Vecuronium 0.02 mg/kg IV	Controlled	Not reported	Taxak [27] 2015	16-60y	i-gel LMA-ProSeal	20 20
Elective short duration surgeries	Phenergan 0.5 mg/kg orally	Atracurium 0.5 mg/kg IV	Controlled	Manometer	Nirupa [12] 2016	2-6y	i-gel LMA-ProSeal	50 50
y = years, LMA = Laryngeal Mask Airway, NMB = Neuromuscular blocker, OLP = Oropharyngeal leak pressure					Liew [14] 2016	21–80y	i-gel LMA-ProSeal	50 LMA-ProSeal
					Das [28] 2017	20-60y	i-gel LMA-ProSeal	50 50
					Banerjee [35] 2018	3-8y	i-gel LMA-ProSeal	35 35
					Singh [29] 2018	18–60y	i-gel LMA-ProSeal	28 28
					Luthra [30] 2019	18-65y	i-gel LMA-ProSeal	20 20
					Oba [16] 2020	<12 months	i-gel LMA-ProSeal	60 60
					Kalra [41] 2021	18–60y	i-gel LMA-ProSeal	50 50
					Shiveshi [3] 2021	2-10y	i-gel LMA-ProSeal	35 35

**Table 2:** Risk of bias assessment for evaluation the quality of each included trials.

Study (author, year)	Random sequence generation	Allocation con cealment	Blinding of participant and personnel	Blinding of outcome assessment	Incomplete outcome data	Selective reporting	Other bias
Singh 2009	Unclear	Unclear	Low	Low	Low	Low	Unclear
Gasteiger 2010	Low	Low	Low	Low	Low	Low	Low
Sharma 2010	Low	Low	Low	Low	Low	Low	Low
Shin 2010	Low	Low	Low	Low	Low	Low	Low
Das 2012	Low	Low	Low	Unclear	Low	Low	Low
Gasteiger 2012	Low	Low	Low	Low	Low	Low	Low
Goyal 2012	Low	Low	Unclear	Unclear	Low	Low	Unclear
Mitra 2012	Low	Low	Unclear	Unclear	Low	Low	Unclear
Van 2012	Low	Low	Unclear	Unclear	Low	Low	Unclear
Chauhan 2013	Low	Low	Unclear	Unclear	Low	Low	Low
Fukuhara 2013	Low	Low	Unclear	Low	Low	Low	Low
Das 2014	Low	Low	Low	Low	Low	Low	Low
Kini 2014	Low	Low	Low	Low	Low	Low	Low
Saran 2014	Low	Low	Low	Unclear	Low	Low	Low
Ekinci 2015	Low	Low	Unclear	Unclear	Low	Low	Low
Jadhav 2015	Low	Low	Low	Low	Low	Low	Low
Kayhan 2015	Low	Low	Low	Low	Low	Low	Low
Henlin 2015	Low	Low	Low	High	Low	Low	Low
Mishra 2015	Low	Low	Unclear	Unclear	Low	Low	Low
Mishra SK 2015	Low	Low	Unclear	Unclear	Low	Low	Low
Mukadder 2015	Low	Low	Unclear	Unclear	Low	Low	Low
Peker 2015	Low	Low	Low	High	Low	Low	Low
Taxak 2015	Low	Low	Unclear	Unclear	Low	Low	Low
Nirupa 2016	Low	Low	Low	Low	Low	Low	Low
Liew 2016	Low	Low	Unclear	Unclear	Low	Low	Low
Das 2017	Low	Low	Low	Low	Low	Low	Low
Banerjee 2018	Low	Low	Unclear	Unclear	Low	Low	Low
Singh 2018	Low	Low	Low	Low	Low	Low	Low
Luthra 2019	Low	Low	Unclear	Unclear	Low	Low	Low
Obs 2020	Low	Low	Unclear	Unclear	Low	Low	Low
Shiveshi 2021	Low	Low	Low	Unclear	Low	Low	Low

### 1. OLP

**Fig 2:**
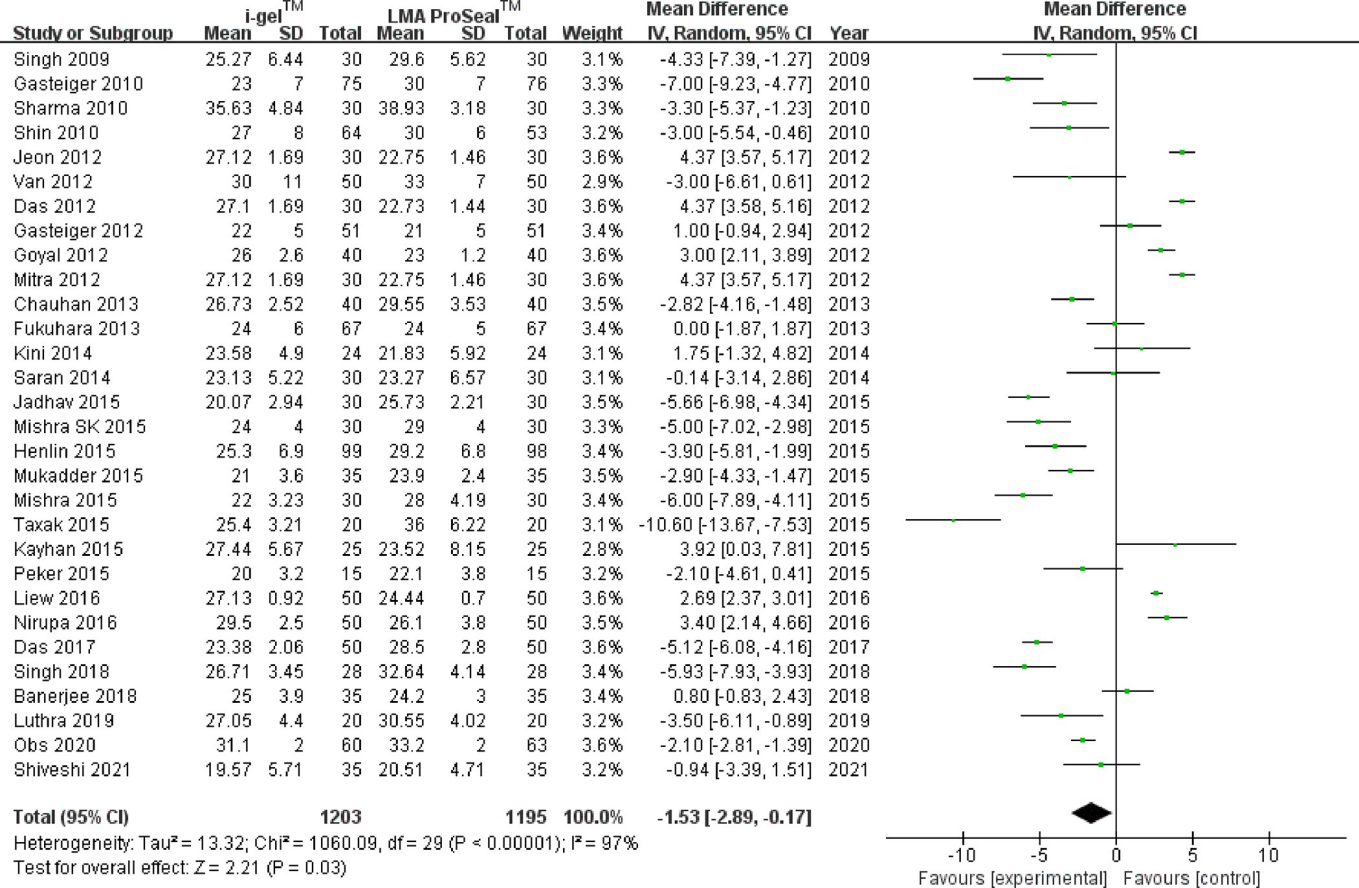
Forest plot for comparison of i-gel^TM^ and LMA ProSeal^TM^ for OLP (cmH_2_O). CI, confidence interval; I^2^, I-square heterogeneity statistic; IV, inverse variance.

**Fig 3:**
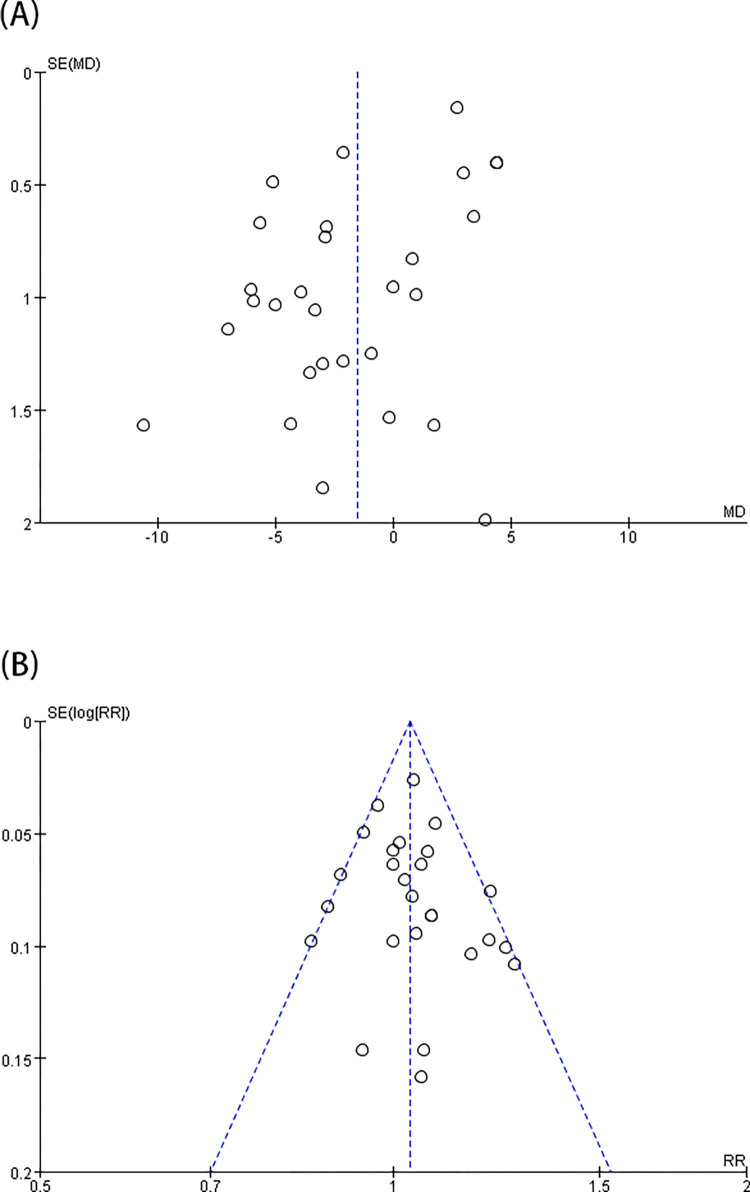
Funnel plots for comparison of i-gel^TM^ and LMA ProSeal^TM^ for OLP (A) and insertion success rate at the first attempt (B).

According to the pooled analysis of data from 30 trials [3, 9–37], i-gel™ offered a considerably lower OLP than LMA ProSeal™ [MD = -1.53 (-2.89, -0.17), *I*^2^ = 97%, P = 0.03] (Fig 2). Upon certification by sensitivity analysis, the pooled result was not altered by a single research. In consideration of substantial heterogeneity, the influence of confounding elements was determined with subgroup analysis (Table 3). According to age subgroup exploration, the pooled outcomes displayed that i-gel™ offered a slightly greater OLP in the children subgroup, although an inadequate statistical difference was observed [MD = 1.34 (˗0.37, 3.04), *I*^2^ = 95%, P = 0.12]; a lower OLP was recorded in the adult subgroup [MD = -3.48 (-5.62, -1.33), *I*^2^ = 98%, P = 0.001] compared with LMA ProSeal™. Considering the potential use of NMB during anesthesia, the pooled results indicated that 15 trials [3, 13, 14, 17, 19–21, 23, 25–29, 33, 35] that applied NMB were covered, and the integrated outcome was lower for i-gel™ than for LMA ProSeal™ [MD = -2.74 (-4.92, -0.57), *I*^2^ = 98%, P = 0.001]. Without NMB, the integrated outcome showed no considerable variation between the two groups [MD = -0.34 (˗2.31, 1.64), *I*^2^ = 97%, P = 0.74]. In case of the pooled analysis of the surgery type, no great difference was found between the two groups with neither laparoscopic nor non-laparoscopic surgery [MD = -1.66 (-6.74,3.42), *I*^2^ = 98%, and P = 0.52; MD = -1.42 (-2.91,0.08), *I*^2^ = 97%, P = 0.06, respectively]. Considering the different measurements of OLP (audible leak and manometric stability), the subgroup analysis showed no great difference between the two groups [MD = -1.55 (-3.45,0.34), *I*^2^ = 97%, P = 0.11; MD = -1.53 (-3.8,0.73), *I*^2^ = 98%, P = 0.18, respectively]. The funnel plot of OLP did not indicate obvious substantial asymmetry (Fig 3).

**Table 3:** Subgroup meta-analysis for oropharyngeal leak pressure with i-gel™ and LMA ProSeal™.

	Subgroup	References	P-value	MD	95% CI	I-square; P-value
age	<18 years	[3, 9–12, 15, 16, 31–35]	0.12	1.34	(-0.37,3.04)	95%;<0.00001
	》18 years	[13, 14, 17–30, 36, 37]	0.001	-3.48	(-5.62,-1.33)	98%;<0.00001
NMB	No	[9–12, 15, 16, 18, 22, 24, 30–32, 34, 36–37]	0.74	-0.34	(-2.31,1.64)	97%;<0.00001
	Yes	[3, 13, 14, 17, 19–21, 23, 25–29, 33, 35]	0.01	-2.74	(-4.92,-0.57)	98%;<0.00001
Laparoscopic surgery	No	[3, 9–12, 14–18, 20–22, 24, 26–37]	0.06	-1.42	(-2.91,0.08)	97%;<0.00001
	Yes	[13, 19, 23, 25]	0.52	-1.66	(-6.74,3.42)	98%;<0.00001
OLP measurement method	Audible leak	[12, 14, 16, 17, 19, 23, 24, 28, 30, 31, 33–37]	0.11	-1.55	(-3.45,0.34)	97%;<0.00001
	Manometer	[3, 9–11, 13, 15, 18, 20–22, 25–27, 29, 32]	0.18	-1.53	(-3.8,0.73)	98%;<0.00001

OLP, oropharyngeal leak pressure; LMA, Laryngeal Mask Airway; NMB, Neuromuscular blocker; MD, mean difference; CI, confidence interval.

### 2. First-insertion success rate, insertion ease of SADs, the time spent on intubation, and gastric-tube first-insertion success rate

**Fig 4:**
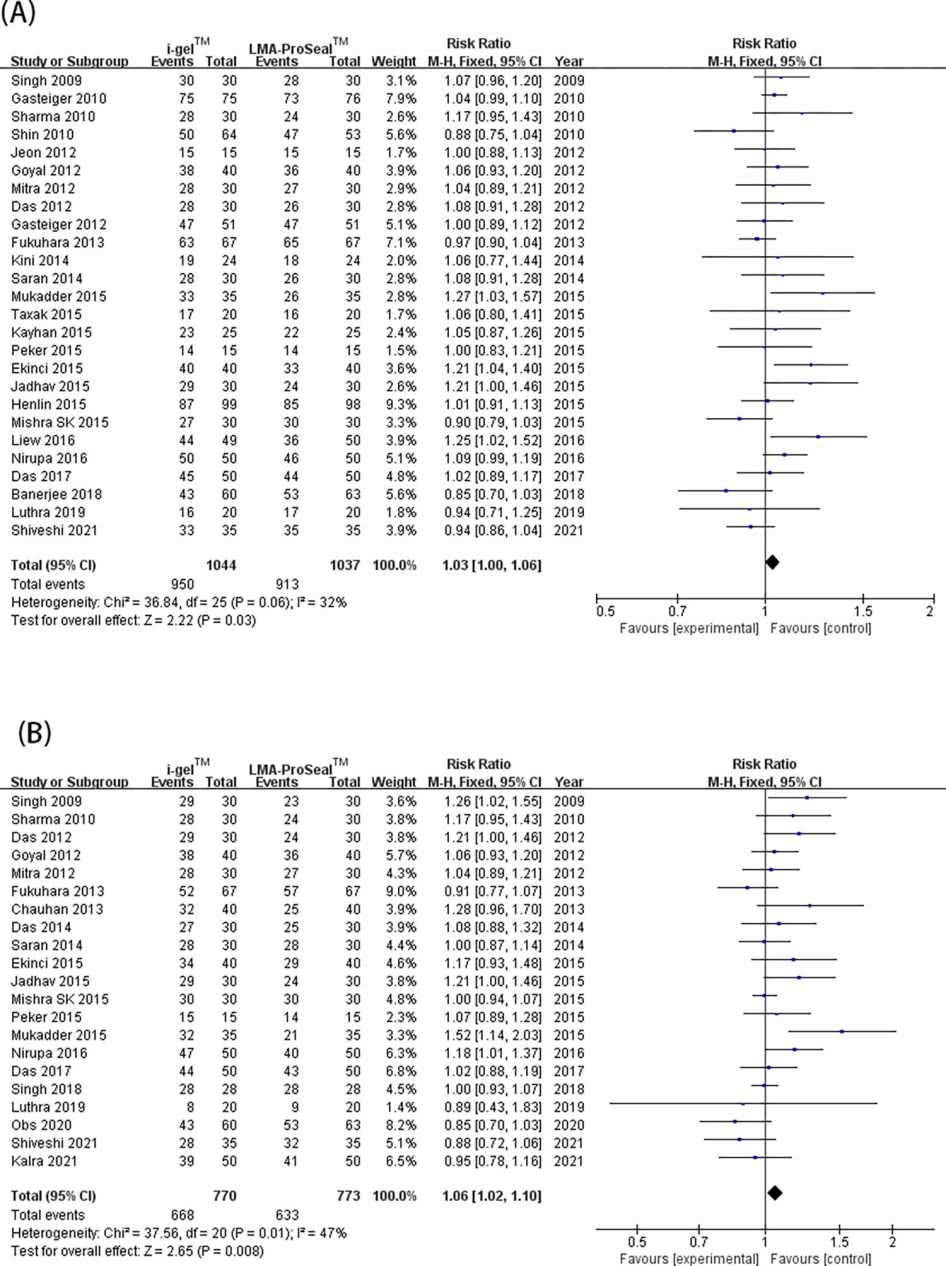
Forest plot for comparison of i-gel^TM^ and LMA ProSeal^TM^ for insertion success rate at the first attempt (A); and ease of insertion (B). CI, confidence interval; I^2^, I-square heterogeneity statistic; IV, inverse variance.

**Fig 5:**
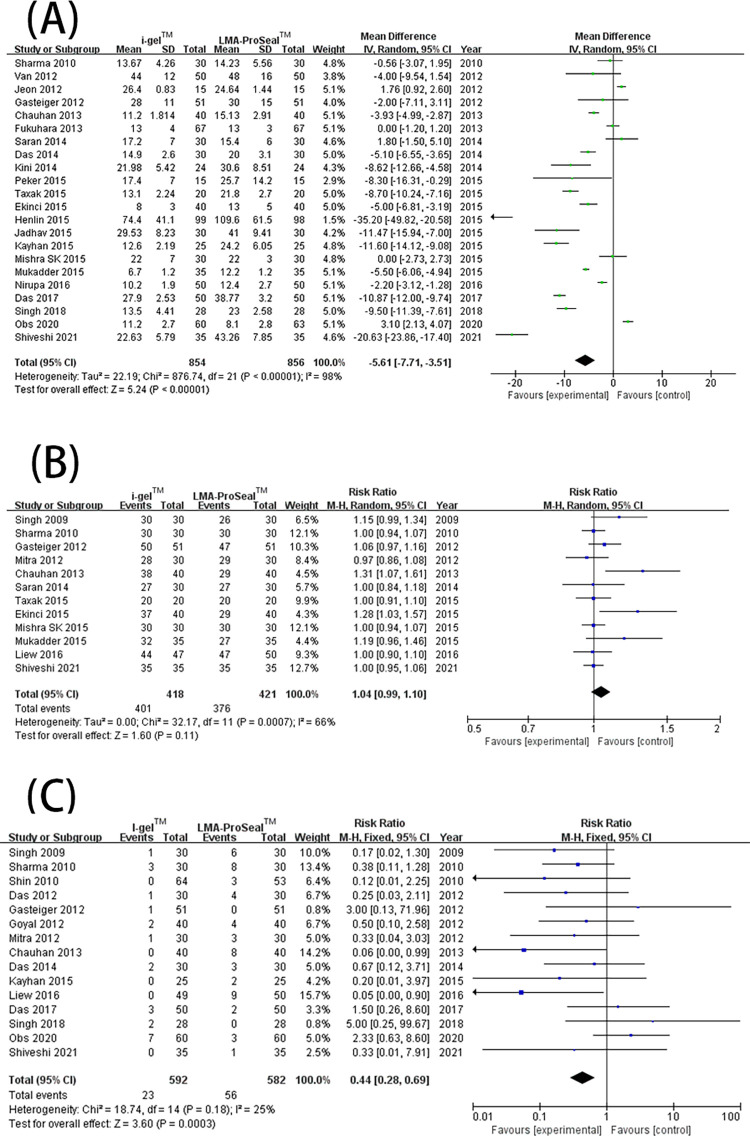
Forest plot for comparison of i-gel^TM^ and LMA ProSeal^TM^ for insertion time (A); gastric tube placement first insertion success rate (B); blood staining on the SADs (C). CI, confidence interval; I^2^, I-square heterogeneity statistic; IV, inverse variance.

**Fig 6:**
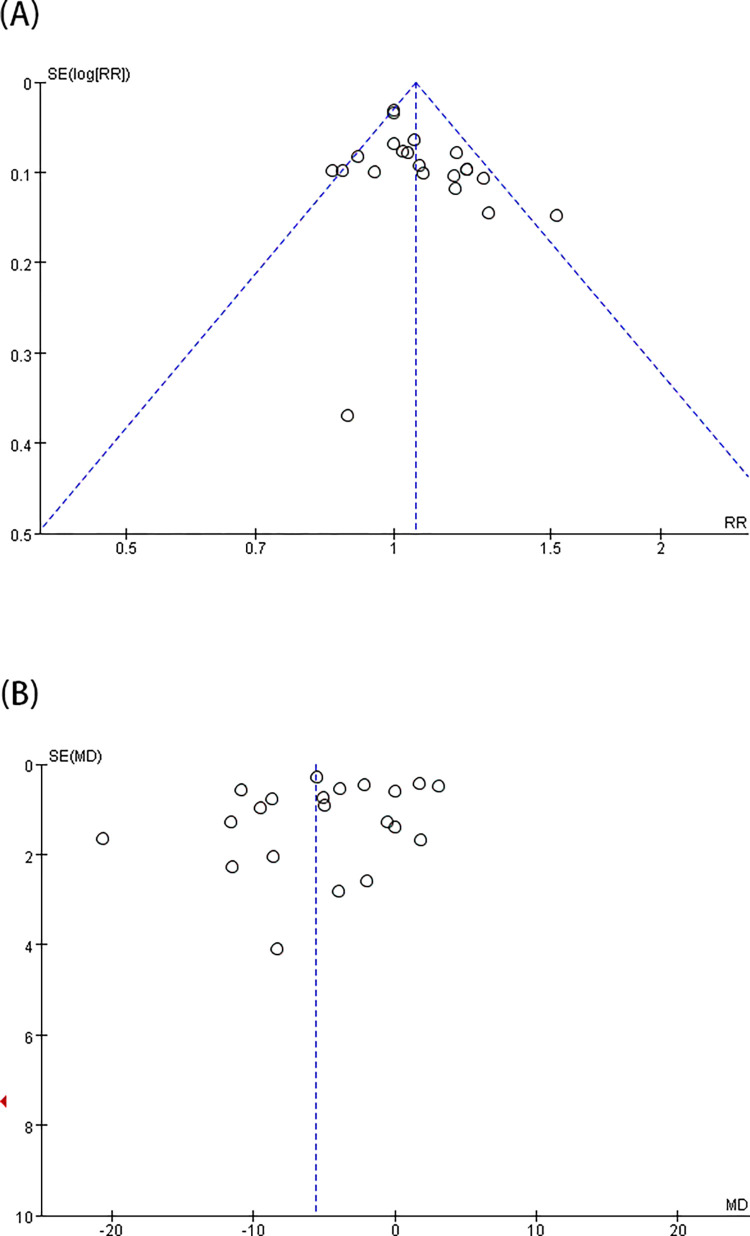
Funnel plots for comparison of i-gel^TM^ and LMA ProSeal^TM^ for ease of insertion (A); insertion time (B).

A total of 26 trials [3, 9–15, 17–20, 22–25, 27–28, 30–36, 40] showed that i-gel™ provided a higher rate of first-insertion success [RR = 1.03 (1.0, 1.06), *I*^2^ = 32%, P = 0.03] than LMA ProSeal™ (Fig 4). Exactly 21 trials [3, 9–12, 16, 17, 19, 21–23, 25, 28–31, 33, 34, 39–41] indicated that the insertion ease was substantially higher for i-gel™ than for LMA ProSeal™ [RR = 1.06 (1.01, 1.11), *I*^2^ = 47%, P = 0.01] (Fig 4). In addition, 23 trials [3, 12–16, 19, 21–25, 27–29, 31–34, 36, 37, 40] showed that SAD intubation time was notably shorter for i-gel™ than for LMA ProSeal™ [MD = -5.61 (-7.71, -3.51), *I*^2^ = 98%, and P<0.00001] (Fig 5). Twelve trials [3, 11, 14, 17, 19, 21, 23, 25, 27, 32, 33, 40] examined the rate of gastric-tube first-insertion success and observed no great difference between the two SADs [RR = 1.04 (0.99, 1.18), *I*^2^ = 66%, and P = 0.11] (Fig 5). With the removal of studies one by one, the heterogeneity of intubation time and the rate of gastric-tube first interpolation success revealed no marked decrease. The funnel plot of first- insertion success rate (Fig 3), insertion ease of SADs, and intubation time (Fig 6) did not indicate obvious substantial asymmetry.

### 3. Adverse events

**Fig 7:**
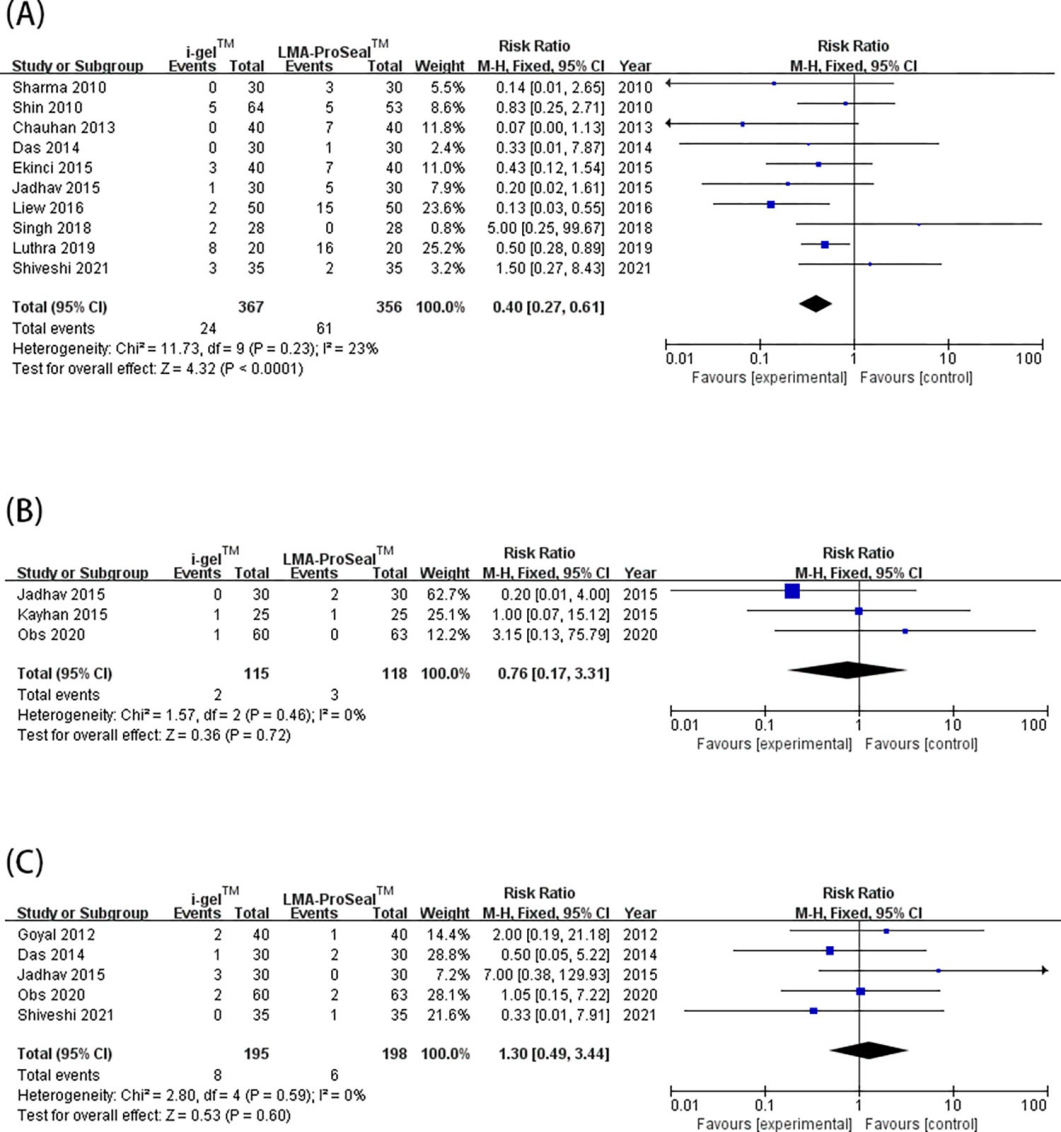
Forest plot for comparison of i-gel^TM^ and LMA ProSeal^TM^ for sore throat (A); laryngospasm (B); cough (C). CI, confidence interval; I^2^, I-square heterogeneity statistic; IV, inverse variance.

**Fig 8:**
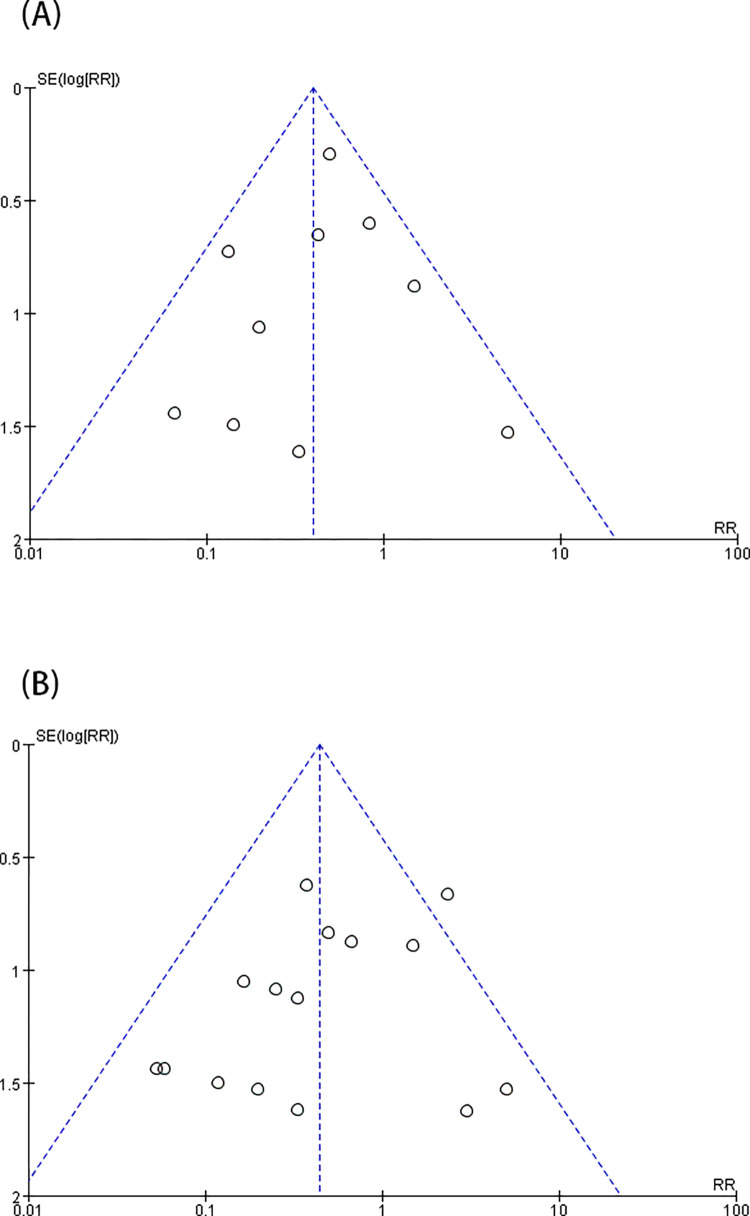
Funnel plots for comparison of i-gel^TM^ and LMA ProSeal^TM^ for sore throat (A); and blood staining (B).

The incidence of revealed adverse events were evaluated: blood staining on the SADs, sore throat, cough, and laryngospasm was shown in 15 [3, 9–11, 14–17, 19–21, 28, 29, 32, 39], 10 [3, 14, 19–22, 29, 30, 39, 40], 5 [3, 10, 16, 22, 39], 3 studies [15, 16, 22], respectively. Blood staining on the SADs after surgery (Fig 5) and sore throat (Fig 7) were greatly more universally occurring with LMA ProSeal™ than with i-gel™ [RR = 0.44 (0.28, 0.69), *I*^2^ = 25%, P = 0.0003; RR = 0.31 (0.18, 0.52), *I*^2^ = 0%, P<0.0001, respectively]. The two groups showed similar incidence of coughs and laryngospasm [RR = 1.17 (0.39, 3.46), *I*^2^ = 0%, P = 0.78; RR = 0.83 (0.15, 4.52), *I*^2^ = 0%, P = 0.83, respectively] (Fig 7). The funnel plot of blood staining did not show evident substantial asymmetry (Fig 8). The included studies reported none of the severe complications.

## Discussion

The major finding of the current meta-analysis is that i-gel™ provided a greatly lower OLP, incidence of blood staining on the SADs, sore throat, and a shorter intubation time than LMA ProSeal™ among patients during general anesthesia. In addition, i-gel™ offered a significantly higher first-insertion success rate and ease of insertion than LMA ProSeal™. No great differences were found in gastric-tube placement first-insertion rate, laryngospasm, and cough between i-gel™ and LMA ProSeal™.

OLP refers to the airway leak or pressure airway sealing, and it is the most significant index for evaluating the security and effectiveness of airway tools [42]. Between the cuff of the mask and soft tissue around the neck was decided the power of the seal [7, 43], the OLP determines the feasibility of the extent of protecting airway and security of positive pressure ventilation. The current meta-analysis observed a greatly higher OLP with LMA ProSeal™ than with i-gel™. The higher OLP in the LMA ProSeal™ group caused by the inflatable cuff with a ventral and dorsal cuff could have led to better seal than i-gel™ with a noninflatable cuff [30]. Growing OLP provides specific merits in fat patients, restrictive and obstructive lung diseases, lithotomy position, and pneumo-peritoneum patients [44].

Patient age, the use of NMB, intra-abdominal pressure during operation, evaluation approach of OLP, and LMA size selection standards may influence OLP [45]. Distinct data heterogeneity in the united OLP outcome was observed in our findings. A great heterogeneity (*I*^2^ = 97%) cannot be reduced although different subgroup analyses were adopted, probably due to the application of various sizes of SADs in these trials. The research by Mitra [11] used a 2.5 device. In Shiveshi’s research [3], despite the use of 2 and 2.5 devices, the device adopted showed the evident size of 2 in more than 70% of kids. In addition, diversities in induction, maintenance, anesthesia depth, measurement standards, and the number of patients researched might also have contributed to the distinct data heterogeneity.

SADs with an inflatable mask show promise in causing tissue distortion, venous compression, and nerve injury, which translate into the growing incidence of related postoperative morbidity [5]. The incrimination of trauma on insertion, various insertions, and pressure brought by cuff against the pharyngeal mucosa cuff volumes and pressure has been made for postoperative complications [46, 47]. In the present study, i-gel™ provided a higher first-insertion success rate, higher ease of insertion, and shorter intubation time than LMA ProSeal™, possibly because of a convenient disposable device, relieve of interpolation by stiff bite block, and the natural oropharyngeal curvature of i-gel™ compared with LMA ProSeal™. In addition, we observed that the application of the i-gel™ is related to a lower incidence of pharyngolaryngeal morbidity (blood staining of the SADs and sore throat) compared with the LMA ProSeal™.

By comparing with a previous review [48], our study presented different findings. First, the included studies in the previous review were published from 2009 to 2014, which is a long time ago. However, nearly 50% of the studies [3, 12, 14–16, 22–30, 34, 35, 40, 41] in our present meta-analysis were published after 2014 and reported conflicting results. Second, this work added several new outcomes compared with the past reviews. The first research showed that i-gel™ can offer a higher first-insertion success rate and insertion ease, similar gastric-tube-placement first-insertion rate, laryngospasm, and cough by comparing with LMA ProSeal™ in adults. Third, previous meta-analyses [49] comparing the two devices reported higher a OLP in i-gel™ than LMA ProSeal™ for pediatric patients, forming a contrast against our findings, which indicated that i-gel™ offers a similar OLP compared with LMA ProSeal™ in children. This disparity may be due to the differences in the included studies. Finally, LMA ProSeal™ did not show a higher OLP compared with i-gel™ under conditions of NMB and laparoscopic surgery.

Several limitations were observed in the current work. First, diversities in induction, maintenance, anesthesia depth, and the number of patients researched might have contributed to the distinct data heterogeneity. In spite of subgroups and sensitivity explorations were performed to control several factors, all possible confounding factors cannot be accounted for. Second, while comprehensively searching the published articles, the bias of potential publication might have been present because of the unsuccess to include in-progress or unpublished studies. Third, the mean difference of OLP from the pooled estimates is 1.53, with the absolute value of OLP from the included studies were all more than 20cmH_2_O. An OLP value of more than 20cmH_2_O is generally accepted as an adequate seal. In clinical practice, the difference in OLP values may not be meaningful, when both devices could achieve a enough seal to provide adequate ventilation. In the end, poor quality was found in several included studies. Two studies [24, 34] conducted a single-blinded rather than a double-blinded trial, and several research did not illustrate the details of binding in the result evaluation. Hence, extra high-quality research and follow-up studies such as trial sequential analysis are necessary to certify our outcomes.

To conclude, our outcomes showed that both i-gel™ and LMA ProSeal™ may offer a good seal to provide adequate ventilation. In addition, i-gel™ offers certain advantages over LMA ProSeal™ (higher insertion success rate at the first attempt, insertion ease, and rapid intubation time) with limited adverse events (blood staining, and sore throat) in anesthetized patients.
