# Correction: Contrast of oropharyngeal leak pressure and clinical performance of I-gel^™^ and LMA ProSeal^™^ in patients: A meta-analysis

**DOI:** 10.1371/journal.pone.0305378

**Published:** 2024-06-06

**Authors:** Yuan Tan, Jingyao Jiang, Rurong Wang

Table 1 is uploaded incorrectly. Please see the correct Table 1 below.

**Table 1:** Characterristics of included trials.

Author/Year	Age	Group	Number	Surgery	Premedication	NMB	Ventilation	OLP measuremen
Singh [17] 2009	adult	i-gel LMA-ProSeal	30 30	Elective hernioplasty, laparoscopic cholecystectomy, tibial plating, humerus plating and skin grafting	Midazolam 1mg IV	Rocuronium 0.9 mg/kg	Controlled	Audible leak
Gasteiger [18] 2010	19-70y	i-gel LMA-ProSeal	75 6	Elective gynaecological or orthopaedic surgery	Midazolam 0.05–0.1 mg/kg orally	No	Controlled	Manometer
Sharma [19] 2010	adult	i-gel LMA-ProSeal	30 30	Elective laparoscopic cholecystectomy	Ranitidine 50 mg IV	Vecuronium 0.08–0.1 mg/kg IV	Controlled	Audible leak
Shin [20] 2010	adult	i-gel LMA-ProSeal	64 53	Lower-extremity orthopaedic surgery	No	Rocuronium 0.6 mg/kg IV	Controlled	Manometer
Das [9] 2012	1-6y	i-gel LMA-ProSeal	30 30	Lower abdominal, inguinal and orthopedic surgery	Midazolam 0.3mg/kg orally	No	Spontaneous	Manometer
Gasteiger [32] 2012	1.5-6y	i-gel LMA-ProSeal	51 51	Short stay elective surgery	Midazolam 0.5 mg/kg orally	No	Controlled	Manometer
Goyal [10] 2012	2-5y	i-gel LMA-ProSeal	40 40	Elective surgeries <1 hour	Midazolam 0.5 mg/kg orally	No	Spontaneous	Manometer
Jeon [13] 2012	18-65y	i-gel LMA-ProSeal	15 15	Laparoscopic gynecologic operation	No	Rocuronium 0.6 mg/kg IV	Controlled	Manometer
Mitra [11] 2012	5-10y	i-gel LMA-ProSeal	30 30	Elective surgeries of less than one hour duration	Midazolam 0.3mg/kg orally	No	Spontaneous	Manometer
Van [37] 2012	18-80y	i-gel LMA-ProSeal	50 50	Elective surgery	No	No	Spontaneous	Audible leak
Chauhan [21] 2013	18-65y	i-gel LMA-ProSeal	40 40	Elective surgery	Alprazolam 0.25 mg orally	Rocuronium 0.6 mg/kg IV	Controlled	Manometer
Fukuhara [31] 2013	3months-15y	i-gel LMA-ProSeal	67 67	Elective surgery	Midazolam 0.5mg/kg orally	No	Not reported	Audible leak
Das [39] 2014	20-30y	i-gel LMA-ProSeal	30 30	Elective surgery	Diazepam 5mg orally	Atracurium 0.5 mg/kg IV	Controlled	Not reported
Kini [36] 2014	18-60y	i-gel LMA-ProSeal	24 24	Elective short surgical procedures	No	No	Spontaneous	Audible leak
Saran [33] 2014	1-12y	i-gel LMA-ProSeal	30 30	Elective short duration pediatric surgery	Midazolam 0.5mg/kg orally	Atracurium 0.5 mg/kg IV	Controlled	Audible leak
Ekinci [40] 2015	18-65y	i-gel LMA-ProSeal	40 40	Elective surgery	Midazolam 0.05 mg/kg intramuscular	Vecuronium 0.1 mg/kg IV	Controlled	Audible leak
Jadhav [22] 2015	18-60y	i-gel LMA-ProSeal	30 30	Short surgical procedures	Midazolam 0.02 mg/kg IV	No	Spontaneous	Manometer
Kayhan [15] 2015	infants and neonates	i-gel LMA-ProSeal	25 25	Elective surgery	Rectal 30 mg/kg paracetamol	No	Ventilated manually	Manometer
Henlin [24] 2015	>18y	i-gel LMA-ProSeal	99 98	Elective procedures	Midazolam 7.5 mg orally	No	Controlled	Audible leak
Mishra [26] 2015	18-65y	i-gel LMA-ProSeal	30 30	Elective surgeries	Midazolam 2 mg IV	Atracurium 0.5 mg/kg IV	Controlled	Manometer
Mishra SK [23] 2015	Adult	i-gel LMA-ProSeal	30 30	Elective gynecological laparoscopic surgery	Midazolam 2 mg IV	Atracurium 0.5 mg/kg IV	Controlled	Audible leak
Mukadder [25] 2015	18-60y	i-gel LMA-ProSeal	35 35	Elective gynecological laparoscopic surgery	Midazolam 0.03 mg/kg IV	Rocuronium 0.6 mg/kg IV	Controlled	Manometer
Peker [34] 2015	1-10y	i-gel LMA-ProSeal	15 15	Extra-ocular ophthalmic surgery	Midazolam 0.3 mg/kg orally	No	Controlled	Audible leak
Taxak [27] 2015	16-60y	i-gel LMA-ProSeal	20 20	Elective surgeries	Alprazolam 0.25 mg orally	Vecuronium	Controlled	Manometer
Nirupa [12] 2016	2-6y	i-gel LMA-ProSeal	50 50	Elective surgeries	Midazolam 0.3 mg/kg oral	Atracurium 0.5 mg/kg IV	Controlled	Audible leak
Liew [14] 2016	21-80y	i-gel LMA-ProSeal	50 50	Elective superficial or peripheral surgery	No	No	Spontaneous	Audible leak
Das [28] 2017	20-60y	i-gel LMA-ProSeal	50 50	Elective short surgical procedures	Midazolam 0.05 mg/kg IV	Vecuronium 0.1 mg/kg IV	Controlled	Audible leak
Banerjee [35] 2018	3-8y	i-gel LMA-ProSeal	35 35	Elective surgeries	No	Atracurium 0.5 mg/kg IV	Controlled	Audible leak
Singh [29] 2018	18–60y	i-gel LMA-ProSeal	28 28	Elective surgical procedures	Alprazolam 0.25 mg oral	Rocuronium 0.6 mg/kg IV	Controlled	Manometer
Luthra [30] 2019	18-65y	i-gel LMA-ProSeal	20 20	Elective short surgical procedures	No	No	Controlled	Audible leak
Obs [16] 2020	<12 months	i-gel LMA-ProSeal	60 60	Minor (<1 hour in duration) elective surgery	No	No	Controlled	Audible leak
Kalra [41] 2021	18-60y	i-gel LMA-ProSeal	50 50	Elective surgery	Alprazolam 0.25 mg oral	Vecuronium 0.02 mg/kg IV	Controlled	Not reported
Shiveshi [3] 2021	2-10y	i-gel LMA-ProSeal	35 35	Elective short duration surgeries	Phenergan 0.5 mg/kg orally	Atracurium 0.5 mg/kg IV	Controlled	Manometer

y = years, LMA = Laryngeal Mask Airway, NMB = Neuromuscular blocker, OLP = Oropharyngeal leak pressure
